# Sharing Experiences in Infancy: From Primary Intersubjectivity to Shared Intentionality

**DOI:** 10.3389/fpsyg.2021.667679

**Published:** 2021-07-14

**Authors:** Henrike Moll, Ellyn Pueschel, Qianhui Ni, Alexandra Little

**Affiliations:** Department of Psychology, University of Southern California, Los Angeles, CA, United States

**Keywords:** primary intersubjectivity, shared intentionality, the second person, social cognition, social development, social understanding

## Abstract

We contrast two theses that make different assumptions about the developmental onset of human-unique sociality. The primary intersubjectivity thesis (PIT) argues that humans relate to each other in distinct ways from the beginning of life, as is shown by newborns' participation in face-to-face encounters or “primary intersubjectivity.” According to this thesis, humans' innate relational capacity is the seedbed from which all subsequent social-emotional and social-cognitive developments continuously emerge. The shared intentionality thesis (SIT) states that human-unique forms of interaction develop at 9–12 months of age, when infants put their heads together with others in acts of object-focused joint attention and simple collaborative activities. According to this thesis, human-unique cognition emerges rapidly with the advent of mind-reading capacities that evolved specifically for the purpose of coordination. In this paper, we first contrast the two theses and then sketch the outlines of an account that unifies their strengths. This unified account endorses the PIT's recognition of the fundamental importance of primary intersubjectivity. Any act of sharing experiences is founded on the communicative capacity that is already displayed by young infants in primary intersubjectivity. At the same time, we question the PIT's interpretation that dyadic encounters have the triadic structure of joint attention. Lastly, we draw on empirical work on the development of joint attention, imitation, and social referencing that serves as evidence that primary intersubjectivity continuously unfolds into the capacity for triadic joint attention.

Human infants reciprocally engage with others from the first few weeks of life. By 6–8 weeks old, they make eye contact, smile at, and summon their partner with cooing vocalizations in face-to-face encounters (Stern, 1977, 1985; Reddy, 2003, 2008, 2011; Trevarthen, 2011). The ability of infants to communicate in this way has been called “primary intersubjectivity” (Trevarthen, 1979). Illustrative descriptions of primary intersubjectivity come from Stern (1977, 1985, 1990), Trevarthen (1979, 1993, 1998), Bråten (2009), Bråten and Trevarthen (2007), Field et al. (1985), Field and Fogel (1982), Cohn and Tronick (1988), Tronick et al. (1978), Reddy (2008, 2011), and others. The following exchange recorded by Stern (1977, p. 3) between a mother and her 3-month-old while nursing exemplifies infants' other-orientation and expressiveness in primary intersubjectivity.

*[…] the mother turned her head and gazed at the infant's face. He was gazing at the ceiling, but out of the corner of his eye he saw her head turn toward him and turned to gaze back at her. This had happened before, but now he broke rhythm and stopped sucking. He let go of the nipple and the suction around it broke as he eased into the faintest suggestion of a smile. The mother abruptly stopped talking and as she watched his face begin to transform, her eyes opened a little wider and her eyebrows raised a bit. His eyes locked on to hers, and together they held motionless for an instant. The infant did not return to sucking and his mother held frozen her slight expression of anticipation. The silent and almost motionless instant continued to hang until the mother suddenly shattered it by saying “Hey!” and simultaneously opening her eyes wider, raising her eyebrows further, and throwing her head up and toward the infant. Almost simultaneously, the baby's eyes widened. His head tilted up and, as his smile broadened, the nipple fell out of his mouth. Now she said “Well hello! …. heelló… heeelloóoo!,” so that her pitch rose and the “hellos” became longer and more stressed on each successive repetition. With each phrase the baby expressed more pleasure, and his body resonated almost like a balloon being pumped up, filling a little more with each breath. The mother then paused and her face relaxed. They watched each other expectantly for a moment. The shared excitement between them ebbed, but before it faded completely, the baby suddenly took an initiative and intervened to rescue it. His head lurched forward, his hands jerked up, and a fuller smile blossomed. His mother was jolted into motion. She moved forward, mouth open and eyes alight, and said*, “*Oooooh… ya wanna play do ya… yeah?… I didn't know if you were still hungry…. no… nooooo…. no I didn't…” And off they went*.

This instance illustrates how both adult and infant contribute to the exchange by taking turns and rhythmically coordinating their responses (Brazelton et al., 1974; Condon and Sander, 1974; Murray and Trevarthen, 1986; Isabella and Belsky, 1991; Rochat et al., 1998; Rochat and Striano, 1999; Bråten, 2009; Trevarthen, 2011). Before much was known about dyadic interaction in great apes, it was speculated that humans inherited the capacity for intersubjectivity from primate ancestors (e.g., Bruner, 1982; Tomasello, 1999). Today's primatological record, however, suggests that apes do not show the same kind of mutual other-orientation that characterizes primary intersubjectivity (Gómez, 1996, 1998; Kano et al., 2012, 2018; Carpenter and Call, 2013). Although non-human primates also pay attention to their conspecifics' faces, they focus less on the eyes than humans do, and, more importantly, do not hold eye-to-eye contact, smile, coo, or make rhythmic movements toward one another (Kano and Tomonaga, 2010; Kano et al., 2012; Grossmann, 2017; Kano and Call, 2017; but see Myowa, 1996, and Ferrari et al., 2006, for reports about mimicry and Bard, 2012, for emotional engagement). There is therefore broad consensus today that primary intersubjectivity is uniquely human (Bruner, 1995; Hobson, 2004; Tomasello, 2019; Bjorklund, 2020). There is, however, disagreement about whether young infants' “protoconversations” (Trevarthen, 1979) with others or whether instead later-developing social-cognitive skills define human sociality and mental development.

Some, most notably Reddy and colleagues, have suggested that primary intersubjectivity is not just essential for social bonding but that it is the source from which all social knowledge and understanding springs (Kaye, 1982; Reddy, 2003, 2008, 2015). In this view, human life is distinctly intersubjective and dialogical from the beginning (at least 2 months onward), and it is this innate intersubjective orientation that defines our human nature. Later forms of sharing experiences that go beyond the dyadic encounter between you and I are, in this account, extended versions of an original “inter-human consciousness” (Rödl, 2021) that is already present in primary intersubjectivity. Call this the primary intersubjectivity thesis (PIT). A different view has been put forth by Tomasello and colleagues in their shared intentionality theory, according to which human-unique sociality develops through a cognitive revolution at 9–12 months of age, when infants engage in new behaviors of joint attention, imitative learning, and cooperative action (Tomasello et al., 2005; Moll and Tomasello, 2010; Tomasello, 2018, 2019). In this view, the kind of social relatedness that defines us as a species–because it transforms the individual intentionality we inherited from our primate ancestors–is one in which our attention to each other is mediated by an object of shared attention or interest, some third entity toward which we orient together. This transformation is enabled by the development of recursive mind-reading processes (“I understand that you want me to attend to x”) that form the cognitive basis of shared intentionality, including joint attention, cooperative communication, and similar cooperatively structured interactions. Call this the Shared Intentionality Thesis (SIT).

The aim of this article is to build a bridge between the PIT and the SIT. There has been relatively little crosstalk between the two theses' proponents, although they share the goal of tracing human-unique sociality to its roots. The SIT has tended to overlook the development of triadic relations from earlier, dyadic, intersubjective relations. More specifically, it has not sufficiently acknowledged that the “sharing” of triadic joint attention is accounted for by the same intersubjective awareness that is already in play when 2-month-olds smile, coo, and express affect in primary intersubjectivity. At the same time, we think that Reddy (2008, 2011), today's main defender of the PIT, has overstated what primary intersubjectivity entails. She suggests that face-to-face encounters between young infants and their caregivers already have the triangular shape of joint attention because the infant experiences herself as the object to which she and her partner are attending. We believe that there is little evidence that young infants mentally step outside of the relation that unites them with the other by considering how they are being perceived by the other. We also think that construing primary intersubjectivity as a compressed version of triadic joint attention underestimates the change that occurs when infants engage in actual, triadic, joint attention (with an object external to self and other), with its ramifications for social learning, perceptivity, and theory of mind.

But there is a tendency toward integration of the two theses. The SIT's ambition is to deliver a comprehensive developmental and evolutionary account of all forms of human-unique relatedness. The SIT has therefore assigned primary intersubjectivity, since its human-uniqueness has been established, a firm place in its theoretical framework as an initial milestone in humans' ontogenetic pathway of social cognition (Tomasello et al., 2005). Young infants' sharing of emotions is argued to serve the purpose of social bonding—a mechanism that is recognized to underly “virtually all forms of uniquely human cooperation and shared intentionality” (Tomasello, 2019, p. 31). The SIT has also modified its evolutionary narrative, the ‘interdependence hypothesis’, to better account for the communicative capacities of even young infants (Tomasello and Gonzalez-Cabrera, 2017).

We want to suggest further integration of what we see as the two theses' strengths: The PIT's recognition of the fundamental importance of dyadic human interchange in early infancy and the SIT's emphasis of the novel quality of later-developing triadic joint attention. Such a hybrid account is not new; it has been suggested by the work of Adamson and Bakeman (1982, 1984), Striano and colleagues (Striano and Rochat, 1999; Striano and Bertin, 2004; Striano and Stahl, 2005) and Hobson (2004). Here, we wish to revive it and give further evidence in its support. In the next part of this article, we articulate on what points we see the PIT and the SIT as differing. In the last part, we discuss some problems of each thesis and broadly sketch the outlines of an account that unifies their strengths.

## 1. Where the Pit and the Sit Differ

We isolate three issues on which the PIT and the SIT differ (see Table 1). The first issue concerns the age at which infants first share experiences with other persons intersubjectively. The PIT maintains that even newborns share experiences with others, whereas the SIT argues that intersubjective sharing begins at 9–12 months. The second difference deals with the issue of whether primary intersubjectivity already has the triadic structure that characterizes joint attention. The PIT affirms this whereas the SIT negates it. The third issue concerns the problem of whether early social-cognitive and social-emotional development is continuous or discontinuous. The PIT states that primary intersubjectivity continuously unfolds into object-centered forms of joint attention over the course of the first year of life. The SIT, by contrast, claims discontinuity, with a sharp onset of joint attention at 9–12 months.

**Table 1:** Three issues on which the Primary Intersubjectivity Thesis (PIT) and the Shared Intentionality Thesis (SIT) differ.

**Issue**	**PIT**	**SIT**
Onset of capacity and motivation to share experiences	By 2 months (primary intersubjectivity)	At 9–12 months (shared intentionality)
Is primary intersubjectivity triadic?	Yes	No
Continuous or discontinuous development	Continuous	Discontinuous

*We draw on the work of Reddy (2008, 2011) for our representation of the PIT's view on the second and third issue. Reddy's thoughts on these issues do not reflect those of other scholars of primary intersubjectivity, such as Stern (1985) or Trevarthen (1978); Hubley and Trevarthen (1979)*.

We shall stress that the PIT's take on the second and third issue represent the ideas of Reddy (2008, 2011). Stern and Trevarthen, pioneers in the study of infant intersubjectivity, do not share Reddy's views on these issues. They both proclaimed discontinuous social development (Stern, 1985, spoke of “quantum mental leaps”) and defined a level or layer of sociality—the “intersubjective self” (Stern, 1985) and “secondary intersubjectivity” (Hubley and Trevarthen, 1979), respectively—that corresponds in timing of onset and content with the SIT's shared intentionality. These authors thus do not claim that dyadic person-to-person engagement already contains all the essential elements of triadic engagement or that the latter is a mere spatial extension of the former. We chose to portray Reddy's ideas on these matters because hers contrasts most clearly with the SIT's and has informed recent empirical investigation (Rossmanith et al., 2014).

### 1.1. Sharing Experiences: When Does It Begin?

For the PIT, dyadic encounters like the one captured by Stern (1977) prove that within 2 months post birth, infants have remarkable relational capacities; they are ready to communicate and share experiences with others. This contradicts the idea that infant and caregiver initially form an undifferentiated bundle from which the infant first needs to separate herself. There is no such task of self-other differentiation, as Stern (1985, p. xiii) notes: “the infant's major developmental task is the opposite one, the creation of ties with others—that is, increasing relatedness.” The PIT's idea is that newborns experience themselves as separate subjects who turn to others in order to create social ties (Rochat, 2011; Rochat and Robbins, 2016; Tasimi, 2020, p. 2). Support for the view that young infants perceive themselves as separate and long for subject-to-subject interchange can be seen in their preference for *high but imperfect* social contingency (Watson, 1972; Murray and Trevarthen, 1986; Gergely and Watson, 1996, 1999; but see Marian et al., 1996). Their preference for highly contingent interaction indicates that they want to engage with subjects like themselves because it is others of their kind that can provide such contingent responses (Meltzoff and Gopnik, 1993; Meltzoff, 2007). Neonatal imitation, if it exists (see Oostenbroek et al., 2016, 2019, and Meltzoff et al., 2018, 2019, for a debate), would provide further and even earlier indication that newborns recognize others as similar and yet different subjects. Infants' rejection of perfectly contingent responses further demonstrates that they do not want to be confronted with a mirror image but strive to interact with someone who is recognizably “other” or different from themselves.

How important positively-toned, rhythmic, exchanges with other humans are for young infants is revealed by how emotionally perturbed they become when their partner abruptly disengages and by the concerted effort they make to reanimate her (Tronick et al., 1978, 1982; Nagy et al., 2017). According to the PIT, all of this shows that even newborns connect with other minds and are aware of others' subjectivity. As Trevarthen and Aitken (2001, p. 4) claim, “the infant is born with awareness specifically receptive to subjective states in other persons.”

The SIT acknowledges that by 2 months of age, infants engage in a kind of “emotion sharing” that helps them and their parent become affectively attuned. But the SIT denies that these exchanges are intersubjective because the infant does not yet recognize others as intentional agents and subjects of experience. Tomasello writes, “Some researchers, especially Trevarthen, believe that these early interactions are ‘intersubjective,’ but in my view they cannot be intersubjective until infants understand others as subjects of experience—which they will not do until 9 months of age” (1999, p. 60). Different criteria are thus invoked to decide if an exchange qualifies as intersubjective. Whereas Trevarthen regards 2-month-olds' participation in protoconversations as sufficient proof that they understand others' subjectivity, thus making the exchange intersubjective, Tomasello demands proof that infants understand self and others as intentional agents. A prerequisite for understanding intentionality, according to him, is that infants experience themselves as instrumental agents, which they begin to do around 8 months of age when they differentiate means from ends in goal-directed activities (Piaget, 1953; Frye, 1991). By 9 months, infants are aware that others are also subjects of intentional and goal-directed action (Woodward, 1998; Cannon and Woodward, 2012)—a realization they allegedly develop by an “argument” from analogy (Tomasello, 1999). Once infants recognize others' intentionality, their motivation and skill for intersubjective sharing sets in. This manifests in a suite of joint attentional behaviors, all of which are said to emerge at 9–12 months (Carpenter et al., 1998). These include:

– perceiving objects together by seeing or hearing them simultaneously or in quick succession and looking back to the partner (e.g., Adamson and Bakeman, 1985; Butterworth and Jarrett, 1991)
– gesturing deictically to objects or events in order to share them (e.g., Liszkowski et al., 2004)
– imitative learning, i.e., re-enacting another's action in recognition of “doing the same” (Hobson and Hobson, 2007)
– turning to other persons as guides by orienting to them when confronted with novel situations (social referencing; e.g., Campos and Stenberg, 1981)
– playing one's part in simple collaborative projects or games with shared goals, such as simple games of give and take (e.g., Carpenter et al., 2005; Tomasello and Carpenter, 2005)

Many of these behaviors are shown just for the sake of sharing, which infants, so long as they do not have autism, find rewarding (Kasari et al., 1990; Gómez et al., 1993; Gangi et al., 2014; Siposova and Carpenter, 2019). Other behaviors are performed to get another to do something (imperative pointing) or to learn how to handle an unfamiliar situation (social referencing).

In their longitudinal study of infants between 9 and 15 months, Carpenter et al. (1998) found that these skills emerge rapidly, are correlated with one another and are all in place by 12 months. The SIT explains the simultaneous development of joint attentional behaviors with a common psychological cause: a socially recursive mind-reading mechanism (“I understand that you intend for me to share this goal/perception”) that adapts infants for mental coordination with other persons, especially adults (Tomasello et al., 2005; Tomasello and Gonzalez-Cabrera, 2017; Tomasello, 2019). The new social-cognitive mechanism has a dual-level structure that represents both the sharedness of the goal and the individuality of the roles and perspectives of the participants (Tomasello, 2019, 2020). In this picture, genuine intersubjectivity begins with a new form of relational thinking that transforms great ape intentionality into the capacity to knowingly act as part of a plural subject (a “we”) in the context of joint attention, cooperative communication, and collaborative action. Only now is there a “meeting of minds” (Bruner, 1995) because only now do infants understand others as subjects of individual and shared experience.

As work by Mundy and others has shown, such a meeting of minds is difficult to realize for infants with autism spectrum disorder because their natural proclivity to establish joint attention is impaired. The greater the impairments in joint attention, the more severe the symptoms of the disorder tend to be (Sigman et al., 1986; Kasari et al., 1990; Mundy et al., 1994). Deeper investigations into the problem of joint attention in autism revealed the importance of breaking joint attention down into the mechanisms of *responding* to others' bids for joint attention (RJA) vs. *initiating* joint attention (IJA). It is particularly the latter capacity, IJA, that is defective in autism (Mundy et al., 2007). These clinical observations dovetail with the SIT's view that triadic joint attention characterizes human social cognition and is decisive for healthy, species-typical, development.

### 1.2. The Object Within: Is Primary Intersubjectivity a Case of Joint Attention?

One of the PIT's main charges against the SIT is its fixation on joint attention *to objects outside of the dyad*, i.e., to physical things other than the interaction partners themselves (Reddy, 2011, p. 141). Reddy (2003, 2008, 2011) argues that we have to look for the first objects of joint attention within the dyad itself, not at a distance. According to her, infants' understanding of the aboutness or object-directedness of attention begins in primary intersubjectivity. The 2-month-old is aware that she is the object of her interaction partner's attention. She experiences the other's gaze on her: “the infant feels the other attending to the self, the infant experiences the relation between looker and object” (Reddy, 2011, p. 144). The encounter with another human is thus the birthplace of self-awareness as much as it is the birthplace of other-awareness. Infants express their budding self-awareness in coy smiles, shy reactions, and other signs of (proto-)embarrassment like looking down or turning away when others look at them. This would imply that primary intersubjectivity is not just a two-place relation connecting you and I, but a three-place relation, with “me” as the object of your individual or our joint attention (I-You-Me). Reddy assumes that the third pole that characterizes joint attention is already present in what is typically thought of as just a dyadic, person-to-person, encounter.

Reddy (2008) thus believes that infants already understand others' intentional states, including their attentional states, by 2 months of age. There is no reason, according to her, to limit our interest to cases of joint attention with distal targets, as the SIT does. Rather than waiting for infants to refer to objects outside of the dyad, we should look for joint attention within the dyadic exchange, in which the infant experiences herself as the target of the other's attention. Primary intersubjectivity has the same object-directedness and therefore the same triangular shape as do cases of joint attention with external referents.

The SIT denies that primary intersubjectivity is a triadic relation or a form of joint attention (Tomasello et al., 2005). Face-to-face interactions between young infants and others are dyadic, not triadic, because they have no topic. There is no common project, goal, or object of interest that unites the participants. There is nothing over which two minds come together. But such a common topic, focus, or goal is what joint attention is all about: that two people knowingly co-orient toward something in the world. In primary intersubjectivity, I orient toward you, and you orient toward me. We are in mutual attention and what you do affects me and vice versa, but there is no shared goal, perception, or action. For the SIT, joint attention serves the purpose of mental coordination that is necessary for joint agency, which is made possible by socially recursive mental processes in which we both have the other as cooperative partner in mind. This enables effective collaboration and communication, including knowledge transmission between generations (Tomasello, 2019). Primary intersubjectivity, although it brings the other psychologically closer to me, does not support cooperative action because it does not bring the world into our shared view. Primary intersubjectivity and triadic joint attention are thus distinct phenomena.

### 1.3. Continuity or Discontinuity?

The PIT argues that social understanding unfolds continuously throughout infancy. Intersubjective attention sharing can be observed in infants as young as 2 months, marking the beginning of gradual growth in human social understanding. What changes over time are the objects of shared attention and the means by which they are shared. As stated in the previous section, Reddy (2008, 2011) argues that 2-month-olds experience themselves as objects of their interaction partner's attention. At 4 months, infants direct the other's attention to their bodies by calling on the other to repeat physical games such as tickling. By 6 months, infants are believed to sense when the other is attending to particular parts of their body (e.g., their feet) or to particular actions they perform (e.g., kicking). From around 7 months, infants direct and manipulate the other's attention by clowning, showing off, and teasing (Reddy, 1991). From 9 months onward, distal targets are rendered into objects of joint attention by way of holding them up, showing, vocally referencing or pointing to them. By 15 months, infants refer to absent entities, such as objects that are typically present but currently missing from the indicated location (e.g., an empty jar). All the while, the infant not only responds to others' bids for attention (RJA) but also initiates joint attention (IJA), to revoke Mundy's distinction of two dissociable processes (Mundy and Newell, 2007).

In support of the continuity claim, Reddy and colleagues conducted a microanalytic study (see Kaye, 1982, for microanalysis) on the development of joint book reading between mothers and their young infants (Rossmanith et al., 2014). Even 3-month-olds are said to have shown nascent abilities to jointly attend to books with their caregiver. What gradually changed with age were the modes of sharing and which aspect of the books was brought into focus. After manually exploring the books' materiality, the dyads shifted their attention to the pictures and their symbolic content, to which infants started to refer gesturally and vocally. The authors infer that, “rather than appearing suddenly supposedly mediated by a newly emerging capacity for joint attention these changes can be seen as part of a gradual development […] coming out of the interplay of multiple strands of development in interaction with the social and cultural environment and the entire ecology of the activity” (p. 18). A similar interpretation is suggested by a dynamic systems perspective that highlights how infants' expanding sensorimotor repertoire (e.g., decoupling of hand and eye movements) and parents' continual adjustments to these changes drive the formation of joint attention and its changes over time (Deák and Triesch, 2006; Triesch et al., 2006; Deák et al., 2013; de Barbaro et al., 2013). The PIT sees this as evidence that joint attention does not suddenly spring into existence by means of a social-cognitive revolution between 9 and 12 months. There are no breaks, leaps, or revolutions in the development of joint attention: *natura non-facit saltus*. A problem that defenders of the SIT would see with these studies is that they leave open whether the infant in fact experienced the interactions with the object as shared. Because no communicative expressions such as sharing looks were reported, this crucial question is left unanswered. And because the studies involve no experimental manipulation of the social setting, it also remains uncertain how infants' object engagement was affected by their partner's actions. In the next section, however, we will report experimental data (surveyed, e.g., by Hoehl and Striano, 2013) that corroborate the PIT's continuity claim.

The SIT acknowledges that protoconversations between young infants and their caregivers are “deeply social in that they have emotional content and turn-taking structure” (Tomasello, 1999, p. 59), and that they serve to create a sense of connectedness and attunement. In a recent iteration of the SIT, Tomasello concedes that affective exchanges between infant and parent are “foundational to virtually all forms of uniquely human cooperation and shared intentionality” (Tomasello, 2019, p. 31). However, these early dyadic exchanges are, in this account, not underpinned by the same psychological infrastructure that supports the joint attentional skills of 9–12-month-olds (Tomasello, 1995). This infrastructure emerged/emerges rapidly both in evolution and ontogeny. A “radically new psychological process” (Tomasello, 2019, p. 15) is said to have transformed the minds of *homo* about 400,000 years ago, just like it transforms the minds of every generation of modern human infants as they are approaching their first birthdays. Overall, the SIT grants that primary intersubjectivity marks a crucial first step in the ontogeny of human-unique social development, but it rejects the idea of a continuous path leading from dyadic intersubjectivity to triadic joint attention. For the SIT, triadic joint attention at 9–12 months is a new phenomenon with a unique social-cognitive base (Tomasello, 1995). Triadic joint attention, not dyadic face-to-face interaction, is responsible for infants' introduction into the world of culture and is therefore principally involved in those processes of cultural transmission and cultural evolution (imitation, pedagogical learning etc.) that define our human nature.

## 2. Toward a Unified Account: From Dyadic Interaction to Triadic Engagement

We now sketch an account that integrates insights from the PIT and the SIT. It is the PIT's merit to have generated persuasive evidence that even young infants participate in reciprocal, dialogical exchanges with others. There is, we think, no reason to deny the intersubjective quality of these exchanges. However, we resist Reddy's attempt to collapse the third pole of triadic joint attention into dyadic infant-caregiver exchanges. We believe that the SIT is right in insisting that primary intersubjectivity is dyadic and that joint attention is a qualitatively different, triadic, relation. There is no convincing evidence that the infant in primary intersubjectivity contemplates the other's perception of herself and has anything further than just the other in mind. At the same time, studies on the development of joint attention, imitation, and social referencing have challenged the SIT's view that the transition from dyadic to triadic engagement occurs as a sudden leap at 9–12 months and instead suggests a gradual process in the second half of the first year of life. We now turn to these issues one by one in the following subsections.

### 2.1. Human Other-Orientation and Its Significance for Development

The PIT has convincingly shown that young infants are remarkably relational and other-oriented. Primary intersubjectivity is the first empirical demonstration of the fact that humans are a relational or transactional species. As Rödl (2014) puts it, humans are, as a species, “one toward another.” He argues philosophically that humans' mutual other-orientation is logically prior to their ability to act as a dual or plural subject. What scholars of primary intersubjectivity have shown empirically is that humans' mutual other-orientation is temporally prior to dual or plural agency as well. Before infants can form a “we” with others and engage in joint attention and joint action, they first must recognize and address others as “you” in dyadic exchanges. Buber (1924) articulates this idea when he remarks that “in the beginning is relation”—with “relation” being his term for the dyadic encounter.

The SIT recognizes the fundamental importance of protoconversations as an important first step for infants and caregivers to bond. As mentioned, the SIT added a corollary to its evolutionary narrative, the “interdependence hypothesis,” such that the emergence of primary intersubjectivity in hominin infants is now intelligible as an adaptation to a cooperatively organized breeding system (Tomasello and Gonzalez-Cabrera, 2017). In its older version, the hypothesis stated that selective pressures to develop collaborative foraging strategies explain the emergence of joint intentionality in human phylogeny and ontogeny. But because infants cannot participate in collaborative hunting, the early onset of the joint attentional capacities subserving such acts seemed mysterious (Hrdy, 2009, 2016). To account for this problem, the new version of the hypothesis argues that humans evolved special social skills not only in response to pressures to create collaborative hunting methods but also cooperative breeding practices. Within this adjusted theoretical framework, the expressive and communicative skills even of young modern infants can be explained by the advantage of eliciting care and attention from their multiple caregivers (Tomasello and Gonzalez-Cabrera, 2017).

But despite these adjustments, the SIT has not fully acknowledged the primacy of humankind's dyadic nature or mutual other-orientation, as is shown by its denial that young infants' exchanges with others are intersubjective. Tomasello's (1999) requirement that infants must be instrumental agents who also attribute instrumental agency to others seems too strong. It is not clear why instrumental agency should matter for the recognition of others as subjects, and why it should not suffice that young infants express a desire for socially contingent interaction with others of their kind (Brazelton et al., 1974).

The SIT emphasizes that joint attention is not the sum of two coinciding acts of attention but a single act of two who know of the jointness of their endeavor: the sharedness of their experience is open between them or mutually transparent (Taylor, 1985; Eilan et al., 2005; Gilbert, 2007; Zahavi, 2015; Siposova and Carpenter, 2019). To confirm that infants engage in joint attention, researchers look for “sharing looks” and “knowing smiles” (Hobson and Hobson, 2007; Carpenter and Liebal, 2011), which are precisely those communicative means infants in primary intersubjectivity deploy to signal their relatedness to their partner. In short, it seems that what puts the sharing into joint attention is the same mutual other-orientation that is already in play in primary intersubjectivity (see also Hobson and Hobson, 2011). We thus agree with the PIT that intersubjectivity is present within mere weeks after birth and that the other-orientation even young infants display in dyadic encounters is what allows for the sharing of experiences in joint attention.

### 2.2. Primary Intersubjectivity Is Dyadic, Not Triadic

Here we critically evaluate Reddy's (2003, 2008, 2011) analysis of primary intersubjectivity as an early form of joint attention. Reddy states that the young infant is not just attending to her interaction partner but that she is simultaneously aware of being the object of her partner's attention. In this conception, the infant's self is doubled: she is subject (“I”) and object of experience (“me”). The infant's awareness of the other's gaze on her is expressed in alleged responses of shyness and coyness (Reddy, 2011, p. 146). This interpretation is, in our mind, overly complex, and infants' other-oriented attention in primary intersubjectivity does not, we think, warrant the interpretation that their attention is flexed back onto themselves in the way Reddy argues.

Reddy cites Buber's (1924) I-Thou mode of engagement in the context of her descriptions of primary intersubjectivity. But Buber in fact stresses that the other is *not* available as an object of empirical experience in an I-Thou encounter. The I-Thou forecloses any kind of objectification of one another because both participants, in Buber's view (1924), give themselves to the other entirely so that each has no object in front of them. If the infant experienced herself as the object of another's attention, she would not stand in an I-Thou relation à la Buber, but she would figure as “it” in what Buber calls the I-It mode of engagement. Reddy's description of how the infant feels the other's gaze on her is more in line with Sartre's idea that self-awareness is born from the embarrassment or shame we feel when we sense that we have been detected or exposed (Zahavi, 2014). But the one who detects and exposes us, even if only in our imagination, is someone who looks at us from a detached perspective, not someone we encounter in mutual recognition (I-You). It thus seems impossible to preserve the I-You character of primary intersubjectivity while also arguing that the infant experiences herself as object of another's attention. We believe it is mistaken to point to humans' embodiment or corporeality (Leiblichkeit) and argue that when humans encounter each other, their mutual attention is mediated by their awareness of being physical objects of attention for one another, thus turning the encounter from a two-place relation into a “three-or-more-place” relation.

According to our more straightforward interpretation, primary intersubjectivity is a dyadic encounter in which infants reach out to a person communicatively with the goal to connect with her, subject-to-subject. There is nothing triadic about this because, as Hubley and Trevarthen (1979, p. 58) write, “this type of interaction is devoid of interest in events or objects in the external situations, or in the activities of either or both partners on objects.” This leaner interpretation is not only more compatible with Buber's view of the human encounter that Reddy wants to endorse. It also reflects more accurately infants' unrefracted other-orientation, rather than preoccupation with themselves, during primary intersubjectivity.

The dyadic exchanges between infant and parent are open to being structurally enriched and expanded into triadic relations over time, allowing for the introduction of objects to which infant and adult attend together, “however slightly these objects are detached from the child's self” (Werner and Kaplan, 1967, p. 43).

### 2.3. Turning Together to the World: The Importance of Triadic Joint Attention

One effect of the PIT's interpretation of primary intersubjectivity as joint attention is the underestimation of actual triadic joint attention and its role as a difference-maker for the child's cognitive development, including language learning, (other forms of) imitative learning, theory of mind, and collaborative action. Longitudinal studies have revealed that joint attention at age 1 predicts concurrent and later language proficiency, both in typically-developing toddlers (Tomasello and Todd, 1983; Tomasello and Farrar, 1986; Kristen et al., 2011; Salo et al., 2018) and in those with autism (Mundy et al., 1990, 2007). Joint attentional capacities at 1 year also predict positive social outcomes, such as social competence in toddlerhood (Van Hecke et al., 2007). Skillful participation in joint attention is furthermore related to theory of mind development. Sodian and Kristen-Antonow (2015) found that declarative pointing at 1 year old predicts belief understanding at age 4.5 years. Similar correlations between joint attentional abilities in the second year and theory of mind at 3 and 4 years have been reported for both typical-developing children (Nelson et al., 2008) and children with autism (Charman et al., 2000).

Moll and colleagues found that infants in the second year who shared objects with others in joint attentional engagement could later discern which objects were (and were not) familiar to the other person. This discriminatory capacity collapsed, however, if infants did not share their experience of the objects (Moll et al., 2007, 2008), suggesting that joint attention is a *sine qua non* for infants' budding understanding of others' experiences. Consistent with this empirical work, philosophers have argued that triangulation with a mutually engaged other is necessary for the acquisition of the concepts of subjective belief and objectivity (Davidson, 1990; Verheggen, 1997).

There is no indication that these empirical and conceptual connections between triadic joint attention on one hand and language, perspectively, and an understanding of other minds on the other are reducible to the influence of primary intersubjectivity. To our knowledge, no correlations with later language development, perspectivity, and theory of mind have been shown to exist for primary intersubjectivity in the first few months of life. One might counter that the absence of such evidence cannot be interpreted as evidence of absence because studies do not go far back enough in time to include measures of dyadic engagement. Prospective studies on language development, joint attention, and theory of mind indeed rarely involve assessments of face-to-face interaction in early infancy. Our conjecture is that even if such measures were included, associations with later social-cognitive milestones might be difficult to find because primary intersubjectivity shows relatively little variation in timing of onset and—at least initially—in frequency, both between dyads and between cultures (Stern, 1977; Wörmann et al., 2012).

Further indication that the movement from dyadic to triadic interaction is key for healthy cognitive development comes from Williams Syndrome. Children affected by this disorder are highly interpersonally engaged and sociable (Jones et al., 2000; Järvinen-Pasley et al., 2008). They are very motivated to initiate and sustain I-You relations, as their strong inclination to make eye-to-eye contact, smile, and show other affiliative behaviors indicates. And yet, their cognitive development is noticeably impaired, as is shown by atypical and delayed language acquisition (Laing et al., 2002), deficits in visuospatial cognition (Frangiskakis et al., 1996; Gray et al., 2006) and overall cognitive functioning (Howlin et al., 1998). A viable hypothesis is that these shortcomings stem at least in part from a deficit in transitioning from dyadic attention (I-You) to object-oriented joint attention (I-You-It). Indeed, reduced abilities to respond to and initiate joint attention have been observed in young children with Williams Syndrome (Klein-Tasman et al., 2007; Järvinen-Pasley et al., 2008). Data from developmental psychopathology thus also suggest that joint attention yields benefits that cannot be reduced to effects of dyadic, face-to-face, interaction.

To sum up, there is persuasive developmental and clinical evidence that being capable and motivated to triangulate with others around objects and events is critically important for children's development across cognitive domains. Although social development begins with infants' drive to connect with others face-to-face, a crucial further step is needed to benefit from one's social connectedness and learn from others about the world. Joint attention seems to be its own form of sharing experiences—one that builds on primary intersubjectivity without “being contained in miniature within [this] earlier constructed foundation” (Adamson and McArthur, 1995, p. 210). This affords a development whereby infants and their partners are no longer just mutual attenders but become co-attenders who knowingly shift their attention to external objects together. Infants need to loosen their grip on others in exclusively dyadic bouts of mutual attention and learn to relate to others as co-attenders with whom they bring the world into shared view.

### 2.4. Continuous Growth: From Dyadic Encounters to Triadic Joint Attention

We now review developmental studies of joint attention, imitation, and social referencing which indicate that, rather than emerging suddenly by way of a “9-months revolution” (Tomasello, 1999, p. 61), these capacities might develop in a more gradual fashion, such that a continuous path from infants' early dyadic to their later triadic relations with others can be traced.

Infants, from around 5–6 months on, slowly express a greater interest in the physical surround. They now often like to be held or carried facing outward, into the world, rather than chest against chest, and their motor capacities allow for the manual exploration of objects (von Hofsten and Rönnqvist, 1988). Importantly, however, these initial explorations of the physical environment occur in others' company rather than solo, and there is strong indication that they are informed by infants' preceding intersubjective awareness. That infants indeed in the first year of life, and prior to the alleged watershed of 9 months, bring their intersubjective competence to bear on their interactions with objects is suggested by cross-sectional and longitudinal research.

Cross-sectionally, Cleveland et al. (2007) conducted a set of experiments in which an adult either looked back and forth between an object and the infant (Joint Attention Condition) or looked back and forth between the object and the ceiling (No Joint Attention Condition). Subsequently measured looking times suggested that 7- and 9-month-olds processed the object more deeply in the Joint Attention Condition than in the No Joint Attention Condition, as shown by a greater visual preference for a different object—one that was not familiar from the previous interaction with the adult. Deeper object encoding in infants 9 months and younger is also suggested by research measuring event-related potentials in the brain. Greater negative central components were detected when an adult alternated her gaze between infant and object than when the adult produced non-triadic gaze shifts (Striano et al., 2006; Parise et al., 2008).

In a large-scale longitudinal study following infants from 5 to 9 months, Striano and Bertin (2004) found that many infants between 5 and 7 months of age showed joint attentional looks to their interaction partners. These looks increased over time and, by 9 months of age, were often accompanied by smiles. The findings point to an earlier and more gradual development of triadic joint attention than has been proposed by the SIT (see also Striano et al., 2009). Further evidence that a budding capacity for joint attention is underway prior to the end of the first year of life comes from a recent longitudinal study with infants from 6 to 10 months (Salter and Carpenter, 2021). The authors set up a test situation in which interesting sights (or sounds) went on and off in bursts behind an experimenter's back but in front of the infant. In this scenario, infants as young as 6 months made active attempts to engage the adult in joint attention, as shown by sharing looks and smiles. The behaviors increased with age, but increases were not significant for any consecutive months, suggesting a continuous growth of joint attention throughout the second half of the first year.

Adamson and Bakeman tracked how dyadic interchanges gradually develop into increasingly demanding stages of joint attention (Adamson and Bakeman, 1982, 1984; Bakeman and Adamson, 1984; Adamson and McArthur, 1995). According to their “forward analysis,” infants in the second year of life get involved in triadic relations first by responding to others' invitations for joint attentional engagement (so-called “passive” or “supported joint engagement”) and then by taking on the role of the instigator by showing, holding up, and pointing to objects (“coordinated joint engagement”). Finally, infants deploy symbolic means of reference to create joint attention (“symbol-infused joint engagement”). While their research suggests a progression from RJA to IJA in Mundy's terms (Morales et al., 2000; Mundy and Newell, 2007), Salter and Carpenter's (2021) findings suggest that infants well under age 1 can initiate joint attention if salient environmental changes entice the infant to share her perceptual experience.

Continuity in the development of triadic relations is also suggested by developmental research on imitation and social referencing. Barr and colleagues traced the development of imitation in the first and second year of life (Barr et al., 1996; Barr and Hayne, 1999). They observed that infants between 6 and 9 months are able to reproduce simple actions they observed in others, such as pulling off a puppet's mitten. If given more opportunities than older infants to watch others' demonstrations, some infants between 6 and 9 months imitated even after a 24-h delay (deferred imitation)—a landmark classically thought to be reached in the second half of the second year. This work points to the presence of at least nascent imitative capacities prior to 9 months of age, with continuous growth of this capacity over time. Research on social referencing also suggests a steady growth rather than a sharp onset of object-oriented joint attention. Walden and Ogan (1988) studied the reactions of infants between 6 and 22 months after their parent made emotionally positive or negative remarks about an ambivalent toy. Infants 10 months and older looked to their parent and aligned their subsequent behavior vis-à-vis the object with their parent's message. Infants between 6 and 9 months showed at least some sensitivity to their parent's expressions by looking toward the parent. Although the study might not satisfy strict criteria for social referencing, according to which infants actively seek information when uncertain (Campos, 1983), it shows that infants are responsive to how others interpret novel situations.

This survey of developmental investigations into joint attention, imitation, and social referencing favors the PIT's view of an earlier and more gradual emergence of triadic relations in infancy. It also corroborates the PIT's stronger emphasis on the affective dimension pervading intersubjective exchanges, dyadic and triadic alike, which has been somewhat neglected by the SIT. The considerations we offer not only support the idea of continuity between dyadic and triadic relations but also appreciate the role of affectivity. Hobson and Hobson (2011) finds the debate of joint attention to be too narrowly focused on the “flash light concept” of attention, which abstracts from the various conative and affective dimensions of our orientations to the world. Infants do not imitate adults' visual fixations of objects but adopt affect-laden attitudes by expressing, like their model, e.g., disgust toward the “yucky” food, disapproval of someone's actions, or amusement by a funny object. Infants' smiles in joint attention (Striano and Bertin, 2004; Salter and Carpenter, 2021) and their responsiveness to the emotional tones in social referencing further support Hobson's idea that infants adopt others' orientations *en paquet*, including their affective qualities (Hobson, 2004; Hobson and Hobson, 2007, 2011). We agree with Adamson and McArthur (1995) who stress that rather than becoming lost in transition from dyadic to triadic exchanges, affective tones become more differentiated and are shared with greater explicitness as infants expand their capacities for joint engagement.

If infants' personal connectedness with their caregivers shapes how they approach objects and situations, as indeed the research suggests, then it would be worthwhile to explore the possibility that the attachment bond—which is forged around the same time as infants begin to bring their intersubjectivity to bear on their engagement with objects—modulates the transition from dyadic to triadic interactions. In contrast to non-human animals, human infants express their attachment not just through proximity-seeking behaviors but through communication (Lyons-Ruth, 2007). Perhaps then infants with suboptimal attachment styles are less inclined to share attention with others. In fact, it has been shown that insecurely attached infants involve others less in joint attention than securely attached infants (Schölmerich et al., 1997; Meins et al., 2011; see also Mohammadzade Naghashan et al., 2021), and that maladaptive strategies classified as disorganized attachment are associated with the lowest levels of joint attention (Claussen et al., 2002).

To conclude, both theoretical and empirical considerations of early social-cognitive and social-emotional development support the PIT's claim of continuity in the development from dyadic to triadic intersubjective exchanges and of the PIT's emphasis of the role of affect in the transactions between infants and adults, whether these transactions are dyadic or triadic.

### 2.5. Summary

In this article, we have contrasted two theses about the ontogenetic beginning of human-specific forms of relatedness and social understanding. One thesis, the PIT, emphasizes the crucial importance of young infants' participation in face-to-face exchanges of affect (primary intersubjectivity), which it regards as the point of origin from which infants' social-emotional and social-cognitive understanding gradually becomes richer and more complex going forward. The other thesis, the SIT, pays relatively little attention to young infants in primary intersubjectivity. Instead, it sees intersubjective relatedness and social cognition as rapidly emerging between 9 and 12 months, when infants begin to share experiences in joint attention. We teased out these accounts' weaknesses and strengths. We agreed with the PIT's criticism of the SIT's relative lack of recognition of young infants' relational capacity and motivation. After all, the same basic communicative competence that infants already display in primary intersubjectivity is “what puts the sharing into joint attention”; and without this sharing, joint attention would not be what it is, as the SIT stresses. We argued that the PIT, by turning the infant in primary intersubjectivity into an object of another's attention, questions the I-You character of the interaction between infant and adult. But we endorsed the PIT's motivation to bridge primary intersubjectivity and joint attention by sketching how one expands into the other in a continuous process, until full-blown forms of joint attention appear around the infant's first birthday. We cited older and more recent empirical research on the development of joint attention, imitation, and social referencing suggesting a more continuous emergence of triadic relations than would be expected by the SIT. In this context, we pointed out that work by Adamson and Bakeman (Adamson and Bakeman, 1982; Bakeman and Adamson, 1984) and Hobson (2004, 2007) adequately captures not only the continuous process with which person-person relations unfold into triadic engagements but also the continued presence and further differentiation of shared affect as infants transition from mutual attention to forming with others a “we” with shared topics of interest and collective pursuits. Future research should be geared to explore this transitional period further, ideally by creating conditions that entice infants to express their desire for sharing experiences.

## Author Contributions

HM laid out the structure and overall argument of the article. She wrote most of the manuscript. EP, QN, and AL were equally responsible for help with specific parts of the manuscript. All authors contributed to the article and approved the submitted version.

## Conflict of Interest

The authors declare that the research was conducted in the absence of any commercial or financial relationships that could be construed as a potential conflict of interest.
